# Perspectives and Experiences of Self-monitoring of Blood Pressure Among Patients With Hypertension: A Systematic Review of Qualitative Studies

**DOI:** 10.1093/ajh/hpad021

**Published:** 2023-02-25

**Authors:** Patrizia Natale, Jia Yi Ni, David Martinez-Martin, Ayano Kelly, Clara K Chow, Aravinda Thiagalingam, Corinne Caillaud, Benjamin Eggleton, Nicole Scholes-Robertson, Jonathan C Craig, Giovanni F M Strippoli, Allison Jaure

**Affiliations:** Sydney School of Public Health, The University of Sydney, Sydney, NSW, Australia; Department of Precision and Regenerative Medicine and Ionian Area (DIMEPRE-J), University of Bari Aldo Moro, Bari, Italy; Nephrology, Dialysis and Transplantation Unit, Department of Medical and Surgical Sciences, University of Foggia, Foggia, Italy; Sydney School of Public Health, The University of Sydney, Sydney, NSW, Australia; School of Biomedical Engineering, The University of Sydney, Sydney, NSW, Australia; The University of Sydney Nano Institute (Sydney Nano), The University of Sydney, Sydney, NSW, Australia; School of Biomedical Engineering, The University of Sydney, Sydney, NSW, Australia; Centre for Kidney Research, The Children’s Hospital at Westmead, Sydney, NSW, Australia; South Western Sydney Clinical School, University of New South Wales, Sydney, NSW, Australia; Department of Rheumatology, Liverpool Hospital, Sydney, NSW, Australia; Ingham Institute for Applied Medical Research, Sydney, NSW, Australia; Sydney Medical School, The University of Sydney, Sydney, NSW, Australia; Westmead Applied Research Centre, Westmead Hospital, SydneyAustralia; Sydney Medical School, The University of Sydney, Sydney, NSW, Australia; Westmead Applied Research Centre, Westmead Hospital, SydneyAustralia; Westmead Clinical School, Westmead Hospital, Sydney, NSW, Australia; The University of Sydney Nano Institute (Sydney Nano), The University of Sydney, Sydney, NSW, Australia; School of Medical Sciences, The University of Sydney, Sydney, NSW, Australia; Charles Perkins Centre, The University of Sydney, Sydney, NSW, Australia; The University of Sydney Nano Institute (Sydney Nano), The University of Sydney, Sydney, NSW, Australia; Sydney School of Public Health, The University of Sydney, Sydney, NSW, Australia; Centre for Kidney Research, The Children’s Hospital at Westmead, Sydney, NSW, Australia; College of Medicine and Public Health, Flinders University, Adelaide, SA, Australia; Sydney School of Public Health, The University of Sydney, Sydney, NSW, Australia; Department of Precision and Regenerative Medicine and Ionian Area (DIMEPRE-J), University of Bari Aldo Moro, Bari, Italy; Sydney School of Public Health, The University of Sydney, Sydney, NSW, Australia; Centre for Kidney Research, The Children’s Hospital at Westmead, Sydney, NSW, Australia

**Keywords:** patient perspective, blood pressure, self-monitoring blood pressure, hypertension

## Abstract

**Background:**

Self-monitoring of blood pressure is a key strategy in managing hypertension but may be challenging and burdensome for patients. The aim of the study was to describe the perspectives and experiences of self-monitoring of blood pressure in patients with hypertension.

**Methods:**

MEDLINE, Embase, PsycINFO, and CINAHL were searched from database inception to March 2022. We used thematic synthesis to analyze the data.

**Results:**

Thirty-five studies involving 872 patients aged 18–95 years were included. Four themes were identified: enabling autonomy and empowerment of own health (allowing access to comprehensive and accurate data, bolstering motivation for lifestyle changes, encouraging diligence in medication taking, gaining interest in self-management, and increasing awareness of health status); providing reassurance and convenience (instilling a sense of security, readiness for troubleshooting, and reducing the frequency of clinical appointments); triggering confusion and stress (anxiety and panic over “bad” numbers, constant reminder of illness identity, disregarded by clinicians, lack of confidence in interpreting and responding to results, redundancy of continuous monitoring, and uncertainties around targets and frequency of measures, concerns of unreliability); financial and operational burden of device (vulnerability preventing use, or unsustainable cost).

**Conclusions:**

Inadequate knowledge about the benefits of lowering blood pressure, home blood pressure monitoring, blood pressure goals, and interpretation of blood pressure values, limited access to home blood pressure monitoring devices, and psychological burden with home blood pressure monitoring limit home blood pressure monitoring.

## Graphical Abstract

**Figure:**
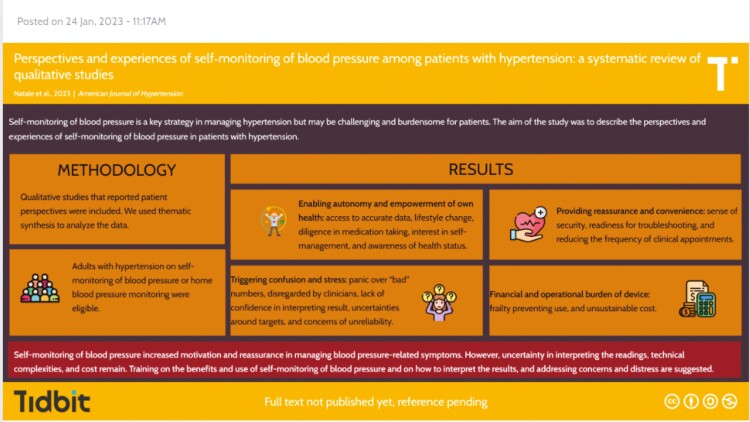
This graphical abstract is also available at Tidbit https://www.editorialmanager.com/ajh/EMDetails.aspx?docid=13418&ms_num=AJH-D-22-00286&sectionID=1#:~:text=https%3A//tidbitapp.io/tidbits/perspectives%2Dand%2Dexperiences%2Dof%2Dself%2Dmonitoring%2Dof%2Dblood%2Dpressure%2Damong%2Dpatients%2Dwith%2Dhypertension%2Da%2Dsystematic%2Dreview%2Dof%2Dqualitative%2Dstudies

Hypertension affects over 1.1 billion people worldwide and is a major risk factor for cardiovascular disease and mortality.^1^ In the past decades, the number of people with hypertension has doubled, and the majority of patients have uncontrolled hypertension.^2^ Guidelines recommend that people with hypertension should check their blood pressure regularly, and only 60% perform self-monitoring of blood pressure.^3^ Guidelines suggest that self-monitoring of blood pressure can improve blood pressure management, but limited evidence is available on the direct effects on long-term outcomes.^4^ Low rates of blood pressure monitoring might be associated with a reduced chance of identifying hypertensive patients with suboptimal blood pressure control.^5^

Patient self-monitoring of blood pressure is a key strategy for managing hypertension, however it can be burdensome for patients.^3^ There have also been concerns about low adherence to self-monitoring of blood pressure, limited accuracy in recording readings, lack of support for vulnerable patients, and financial barriers.^6^ Patients have reported uncertainty about the accuracy of blood pressure readings, complexity in the use, and being unable to interpret readings.^7^

The evidence on patient perspectives on self-monitoring of blood pressure is limited. The synthesis of multiple qualitative studies can generate comprehensive insights on patient perspectives regarding self-monitoring of blood pressure in people with hypertension. We aimed to describe patient perspectives and experiences in self-monitoring of blood pressure, to inform strategies for optimizing the acceptability of self-monitoring of blood pressure.

## Methods

We followed the Enhancing Transparency of Reporting the Synthesis of Qualitative Research framework to report our study.^8^

### Selection criteria

Qualitative studies that reported the perspectives and experiences of adults aged 18 years and over with hypertension as defined by the authors of the included studies (generally defined as blood pressure exceeding 130/80 mm Hg, or those requiring antihypertensive medications^9^) on self-monitoring of blood pressure or home blood pressure monitoring were eligible. Studies that reported epidemiologic or nonprimary studies, quantitative surveys, or reported perspective from health professionals, caregivers, or people without hypertension were excluded. Studies that addressed only ambulatory blood pressure or pulmonary artery pressure measurements were also excluded. Non-English articles were excluded.

### Data sources and searches

We searched MEDLINE, Embase, PsycINFO, and CINAHL from inception to the 8 March 2022 (Supplementary Table S1). We used Medical Subject Headings (mesh) terms and text words related to “hypertension”, “blood pressure devices/monitoring”, and “qualitative studies”. The search strategy and search terms used are outlined in Supplementary Table S1. We searched the reference lists of relevant studies and Google Scholar. Three reviewers independently screened the title and abstracts for inclusion and discarded those that did not meet the inclusion criteria. Full texts were reviewed, and eligible studies were included. Any discrepancies were resolved by discussion.

### Appraisal of transparency of reporting

The transparency of reporting of each included primary study was assessed using an adapted Consolidated Criteria for Reporting Qualitative Health Research (COREQ)^10^ framework. Three reviewers independently assessed each study and discrepancies were resolved after a discussion with another reviewer.

### Data analysis

Thematic synthesis was used to inductively identify concepts.^11^ All participant quotations and text of each study were extracted. Two reviewers coded the data line by line by using HyperRESEARCH (version 4.5.1) and inductively identified preliminary themes and subthemes that captured patient perspectives on self-monitoring of blood pressure. We coded the text from each study into these concepts, creating new concepts, and then categorized similar concepts into broader themes. Investigator triangulation was achieved by discussing the preliminary themes with a third reviewer to ensure the findings captured the full range and depth of the data. We developed an analytical thematic schema to identify conceptual patterns and links among the themes.

## Results

### Literature search and study description

**Figure 1.**
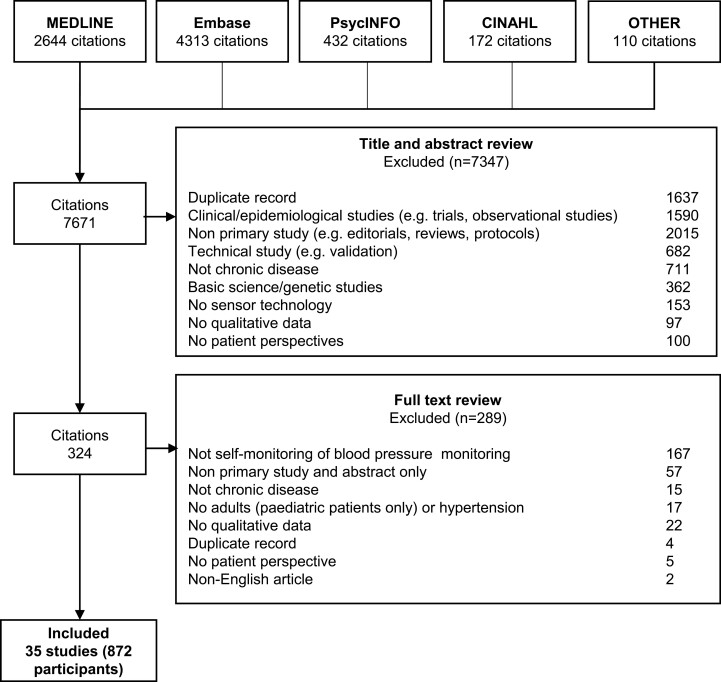
Search results.

We included 35 studies involving 872 patients aged from 18 to 95 years (2 studies did not specify the number of patients) (Figure 1). The studies were conducted across 12 countries. Twenty-two studies conducted semi-structured interviews, 10 used focus groups, and 3 used both interviews and focus groups. The participant and study characteristics of the included studies are shown in Table 1.

**Table 1.** Characteristics of the included studies

Study ID	Country	Population	Age (y)	Sex (*n*)		N	BP monitoring devices	Methodological framework	Data collection	Data analysis	Context, topics, scope
				**F**	**M**						
Abdulla^12^	Malaysia	Hypertension	30–75	NS	NS	24	Home BP monitor	Grounded theory	Focus groups, semi-structured interviews	Grounded theory analysis	Experiences in using self-initiated home BP monitoring
Albrecht^13^	Canada	Hypertension	70–95	4	3	7	Home BP monitor	Qualitative study	Semi-structured interviews	Thematic analysis	Usability and acceptability of home BP telemonitoring
Allen^14^	US	Hypertension	55^*^	23	17	40	Home BP monitor + texting	Qualitative study	Semi-structured interviews	Thematic analysis	Behavior changes from automated SMS-facilitated home BP monitoring program
Al-Rousan^15^	Peru, Cameroon, and Malawi	Hypertension	57.4^*^	41	35	76	Home BP monitor	Qualitative study	Semi-structured focus groups	Thematic analysis	Perceptions and attitudes in self-monitoring BP
Aquino^16^	Canada	Hypertension and high risk for preeclampsia	NS	7	0	7	Home BP monitor + telemonitoring	Qualitative study	Semi-structured interviews	Thematic analysis	Needs and challenges of self-monitoring BP
Bengtsson^17^	Sweden	Hypertension	49–82	9	6	15	Home BP monitor + mobile App	Qualitative study	Focus groups	Thematic analysis	Perspectives of hypertension and its management and development of mobile self-report system
Bostock^18^	UK	Hypertension	NS	6	10	16	Home BP monitor + mobile phone-based telemonitoring	Qualitative study	Focus groups	Thematic analysis	Acceptability of telemetric monitoring of BP
Cairns^19^	UK	Gestational hypertension or preeclampsia	32.2* (2.2)	68	0	68	Home BP monitor	Qualitative study	Semi-structured interviews	Thematic analysis	Perceptions and attitudes in self-management postpartum BP
Carter^20^	UK	Hypertension and preeclampsia	NS	23	0	23	Home BP monitor	Qualitative narrative study	In-depth interviews	Thematic analysis	Perceptions and attitudes in self-monitoring BP
Evangelidis^21^	Multinational	HD and PD patients with hypertension	NS	NS	NS	NS	Home BP monitor	Qualitative study	Focus groups	Thematic analysis	Perceptions and attitudes in self-monitoring BP
Geerse^22^	Finland	Hypertension	NS	1	2	3	Home BP monitor, continuous BP monitoring device	Qualitative study	Semi-structured interviews	Thematic analysis	Experiences informing design of BP monitoring care pathway
Glynn^23^	Ireland	Hypertension	NS	NS	NS	50	Home BP monitor, mobile app	Qualitative study	Focus group	Thematic analysis	Patient experiences of self-management technology for hypertension
Grace^24^	Canada	Heart failure with and without hypertension	NS	NS	NS	14^	Smart-home lab: blanket accelerometer, force sensors in the bed, floor tile, floor electrodes, and chair electrodes and force sensors (respiration, weight, HR, BP, temperature)	Interpretive description	Semi-structured interviews	Interpretive descriptive method	Perspectives of seniors on using smart-home systems to monitor physiological parameters
Grant^25^	UK	Hypertension	49–80	9	7	16	Home BP monitor	Qualitative study	In-depth interviews	Constant comparative method derived from grounded theory	Motivations for self-monitoring BP
Halifax^26^	Canada	Hypertension	55–81	17	7	24	Home BP monitor + telemonitoring	Applied qualitative case study	Focus groups	Thematic analysis	Information needs for the design of a telemedicine for hypertension
Hall^27^	US	Heart failure with and without hypertension	64^*^	5	10	11^	Home monitoring device (BP, weight, glucose, oxygen saturation)	Qualitative study	In-depth interviews	Thematic analysis using constant comparison	Perspectives and experiences of using technology to manage health and heart failure symptoms
Hanley^28^	UK	Hypertension	NS	10	15	25	Home BP monitor + telemonitoring	Qualitative study	Semi-structured interviews and a validation focus group	Thematic analysis using constant comparison	Barriers and facilitators to the use of remote BP telemonitoring
Helou^29^	Multinational	Gestational hypertension or preeclampsia	NS	27	0	27	Home BP monitor	Qualitative narrative study	In-depth interviews	Thematic analysis	Perceptions and attitudes in self-monitoring BP
Jones^30^	UK	Hypertension	49–84 (70*)	10	13	23	Home BP monitor	Qualitative study	Semi-structured interview	Thematic analysis using constant comparison	Perspectives of self-monitoring and self-titrating of antihypertensive medications
Jongsma^31^	Netherlands	Gestational hypertension or preeclampsia	34.2^*^	11	0	11	Home BP monitor + digital monitoring platform	Qualitative narrative study	Semi-structured interview	Thematic analysis	Experience in digital monitoring platform
Koopman^32^	US	Hypertension	59^*^	10	6	16	Home BP monitor	Qualitative study	Focus groups	Rapid qualitative analysis and final thematic analysis	Visual communication of blood pressure readings
Lambert-Kerzener^33^	US	Hypertension	61^*^	27	119	146	Home BP monitor + interactive voice response technology	Qualitative study	Semi-structured interviews	Content analysis and consultative and reflexive team analysis	Experience of participating in a multifaceted hypertension intervention
Lu^34^	Taiwan	Hypertension, diabetes	50–86 (67*)	NS	NS	20	Monitor (BP, glucose) + telehealth	Qualitative study	Semi-structured interviews, focus groups	Content analysis	Consumer experiences of home telehealth
McBride^35^	Ireland	Hypertension	43–74 (62*)	6	5	11	Home BP monitor + self-management app	Qualitative study	Semi-structured interviews	Thematic analysis	User experience of a self-management app
Munyungula^36^	South Africa	Gestational hypertension (preeclampsia)	18–43	14	0	14	Home BP monitor	Qualitative study	Semi-structured interviews	Thematic analysis	Perceptions and attitudes in self-monitoring BP
Ondienge^37^	UK	Hypertension, diabetes	>64	NS	NS	NS	Blood pressure monitor (BP) + device to allow multi-user identification	Qualitative study	Focus groups	Thematic analysis	Perspectives of and experiences with multi-user identification for remote patient monitoring devices
Ovaisi^38^	UK	Stroke with hypertension	47–86 (70*)	9	17	26	Home BP monitor	Qualitative study	Semi-structured interviews	Thematic analysis	Patients experience of BP self-monitoring and nurse let support
Payakachat^39^	US	Gestational hypertension (preeclampsia)	NS	37	0	37	Home BP monitor + mHealth	Qualitative study	Semi-structured interviews	Thematic analysis	Perceptions and attitudes in mHealth
Rickerby^40^	UK	Hypertension	51–67	8	5	13	Home BP monitor	Phenomenology	Semi-structured interviews	Phenomenological analysis	Experiences and opinions of home BP monitoring
Rohela^41^	US	Hypertension	57^*^	21	3	24	Home BP monitor + OWL-H platform	Qualitative study	Semi-structured focus group	Grounded theory analysis	Experiences and opinions of OWL-H platform
Schmid^42^	US	Stroke, transient ischemic attack with hypertension	67^*^	1	27	28	Home BP monitor	Qualitative study	Focus groups	Thematic analysis	BP self-management practices and preferences
Tompson^43^	UK	Hypertension	NS	14	15	29	Home BP monitor, waiting room monitor	Qualitative study	Semi-structured interviews	Thematic analysis	Patient experience of out-of-office BP monitoring
Vasileiou^44^	UK	Hypertension	NS	NS	NS	8	Home BP monitor	Qualitative study	Semi-structured interviews	Thematic analysis	Patient experience of out-of-office BP monitoring
Ware^45^	Canada	Heart failure with and without hypertension	NS	NS	NS	NS	Wireless sensor devices (BP, weight) + smartphone app	Qualitative study	Semi-structured interviews	Content analysis	Experiences of patients with telemonitoring to improve program sustainability and scalability
Xiao^46^	China	Hypertension	59.6 (8.9)^*^	10	10	20	Home BP monitor + mobile app	Qualitative study	Semi-structured interviews, focus groups	Thematic analysis	Blood pressure telemonitoring and its effects on self-care and clinical management

Abbreviations: UK, United Kingdom; US, United States; F, female; M, male; BP, blood pressure; HR, heart rate; HD, hemodialysis; PD, peritoneal dialysis; NS, not stated.

^*^Data reported as mean with or without standard deviation; ^Only patients with cardiovascular disease and hypertension were included.

### Comprehensiveness of reporting

The comprehensiveness of reporting among the included studies is shown in Table 2. Of the 26 possible items included in the appraisal framework, studies reported between 5 and 23 items. Participant selection strategy and participant characteristics were reported in 30 (86%) and 33 (94%) studies, respectively. Nineteen (54%) studies specified theoretical or data saturation (Supplementary Table S2).

**Table 2.** Comprehensiveness of reporting in included studies

Item	Studies reporting each item	Number of studies
Personal characteristics		
Interviewer/facilitator identified	^12,14–16,19,20,28,29,32–35,44,45^	14
Occupation of the interview/facilitator	^13–16,19,20,23,24,26,28,29,32,35,38,40,43,46^	17
Experience or training in qualitative research	^15,16,19,23,28,29,32,35,43,46^	10
Relationship with participants		
Relationship established before study start	^12,15,16,19,23,29,35,39,41,43^	10
Participant selection		
Selection strategy	^12,14–18,20,22,23,25–38,40–46^	30
Method of approach or recruitment	^12,14,16–20,25,26,28,29,31,32,34–36,40–46^	21
Sample size	^12–20,22–31,33–46^	33
No. and/or reasons for nonparticipation	^14,15,19,20,24,27,29,30,35,38,40,41,43,44^	14
Setting		
Venue of data collection	^12–20,23–25,28–31,33,35,39–41,43–46^	25
Presence of nonparticipants (e.g., clinical staff)	^14,19,23,29,35,43,46^	7
Description of sample	^12–19,21–36,38–43,45,46^	33
Data collection		
Questions, prompts, or topic guide	^12–19,22–25,27–36,38,40,41,43–46^	29
Repeat interviews/observations	^14,16,29,35^	4
Audio/visual recording	^12–20,22–31,33–46^	33
Field notes	^12,18,19,22,25,29,30,32,35,36,38,40^	12
Duration of data collection	^13,15,16,19,20,26,28–30,34–36,38,39,43,46^	16
Translation and interpretation	^15^	1
Protocol for data preparation and transcription	^12–15,17–19,22–35,37,38,40,42,43,45^	27
Data (or theoretical) saturation	^12,14–17,19,20,23,25,28–31,35,36,40,43–45^	19
Data analysis		
Researcher/expert triangulation	^12,14–18,20–35,38–46^	31
Translation	^15^	1
Derivation of themes or findings	^12–35,37–46^	34
Use of software	^12–14,16,17,19–21,23–25,27–29,31–33,35,37–39,41–46^	27
Member checking	^16,19,20,23,25,28,31,32,35,43^	10
Reporting		
Participant quotations or raw data provided	^12–25,27–35,37–46^	33
Range of depth of insight into participant perspectives	^12–15,17,19,22,23,25,28,30–32,36,37,39,42,43^	18

### Synthesis

**Figure 2.**
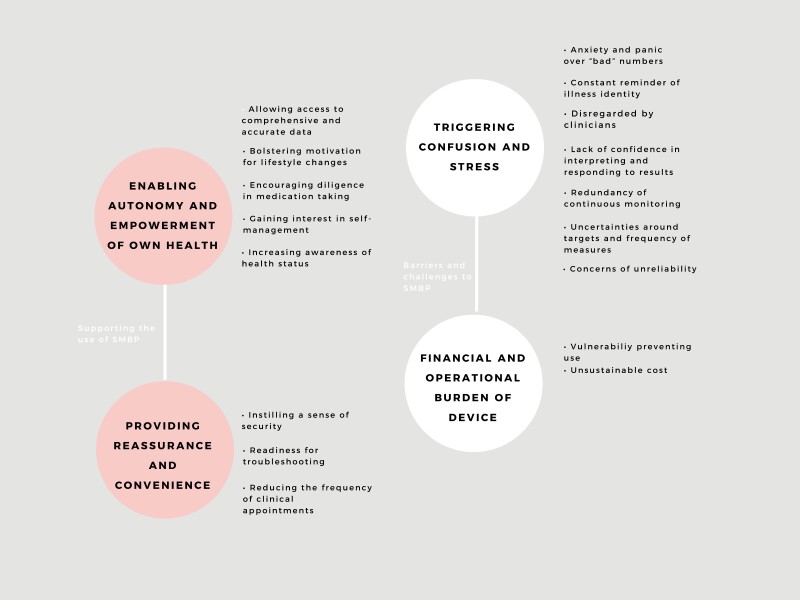
Thematic schema. Abbreviation: SMBP, self-monitoring of blood pressure.

We identified 4 themes: enabling autonomy and empowerment of own health, providing reassurance and convenience, triggering confusion and stress, and financial and operational burden of device. Selected participant quotations to support each theme are provided in Table 3. The conceptual relationships among the themes are depicted in Figure 2.

**Table 3.** Selected quotations for each theme

Theme	Selected illustrative Quotes	Contributing studies
**Enabling autonomy and empowerment of own health**		
** Allowing access to comprehensive and accurate data**	“When you’re doing your blood pressures yourself, you’re just seeing one number at a time. I’m not tracking my averages. I’m just looking at one number at a time. But, having the averages like kind of given to you, it’s like, ‘Oh, okay,’ this is how I’ve been doing the last few weeks. It’s the more significant number, I think.”^14^ “I don’t know if my blood pressure is high or low I have no idea so all I was trying to do was understand because I knew every time I went to the doctor it was up because as soon as you see the doctor you go... he’s taking out the blood pressure thing he is going to say it is up... so by doing it in a more relaxed setting at home I just wanted to see would it be different and it was. It was substantially different, and it appeared to be much lower than every time I went to the doctor.”^23^ “It’s objective data that you get from the machine; what I am telling you it’s very subjective.”^27^	^14,15,18,19,22,23,25,27,28,31,33,34,36,38,40,43^
** Bolstering motivation for lifestyle changes**	“What do I do? I will note down what food I eat and what activity I did. That was during the holidays. I will note down today what did I eat, then I said maybe this food is good for me then I will note down whether I go for a walk in the morning, whether I sweat. I noticed when I sweat the pressure goes better.”^12^ “So, you get an understanding, okay, you’re slowly going up, so you need to, you know, dial back the diet, or push up the exercise, or do something, because it’s going in the wrong direction.”^14^ “Incentive to improve my quality of life would really motivate in using this technology.”^37^	^12,14,28,33,34,37,43,44^
** Encouraging diligence in medication taking**	“It is better (with home blood pressure monitoring) because you can see whether the medicine is effective. Even now when I am on medicine already, before taking the medicine I will go and measure, after taking the medicine I will also measure. That’s why I notice that Coversyl is more effective for me.”^12^ “Because when I’m seeing those high numbers, I’m taking (my BP medication) as prescribed. Before I would just take it any time in the day. Now I take it in the morning, and I take my second one in the afternoon.”^14^ “The device will show an abnormal condition with unstable blood pressure values. It reminds me to think about whether I have taken my medication. Sometimes it is possible to forget to take medicine, but regularly measuring my blood pressure can help me develop the habit of taking my medicines. The advantages include measuring blood pressure regularly, taking medicines on time, and maintaining a stable lifestyle.“^34^	^12,14,18,23,29,31,33,34,38,43^
** Gaining interest in self-management**	“My blood pressure is a little better than I believed that it was, and then being that it was better, and I was proud of myself, it made me want to keep it like that, or go a little lower, so I’ve been slowly cutting out a few things.”^14^ “I think it’s important for me as a patient to be more intentional and more in, participating and not be so passive with my healthcare.”^32^ “I see it (self-monitoring) as a way of trying to convince the GP to take me off it (antihypertensive medication).”^43^	^14,15,19,21,31,32,34–36,41,43,46^
** Increasing awareness of health status**	“When they take my blood pressure, I didn’t know what the numbers meant at all and now I know what they mean so that’s helped me a lot because you know they check my blood pressure, ok, I don’t know if it’s normal. It’s high, it’s low, so I started asking question and now I know what it means so that helped me a lot.”^33^ “But when you’re taking your blood pressure yourself, you have a hold on what’s going on. I can’t explain it very clearly; the words just don’t seem to come at the moment, but you feel that you have some knowledge of what’s going on. I think that’s it. I know the doctors always like, say, ‘Have you any questions?’ but the thing is, you can’t really think of many questions when you’re there. It’s only about 3 weeks later that something crops up! (little laugh) But mostly I do feel that most of the doctors do explain clearly what they’re going to do or what they’re expecting. But having a finger in the pie, helps (laughter)!”^38^ “Maybe your blood pressure is high then, if you can monitor it at home, then you’ll be alert and go seek help at the clinic.”^36^	^14–17,19–21,26,31,33–39,46^
**Providing reassurance and convenience**		
** Instilling a sense of security**	“I am reassured, and I feel quite happy with the fact that I know that my blood pressure is ok. I don’t have to think ‘oh gawd I haven’t been to the doctor for 4 months; I wonder if me blood pressure is alright?’ I know it is.”^30^ “... it’s a safeguarding away... I get peace of mind to know that my blood pressure is more or less they say on a level.”^25^ “It’s reduced my anxiety and put me back in control.”^19^	^12,15–17,19–21,24–26,30,33–36,38,39^
** Readiness for troubleshooting**	“So usually it’s like that (checking irregularly). Except when I feel very bad, like something is wrong. Sometimes I may feel some chest pains then I’ll go and check.”^12^ “I am responsible for keeping track of my blood pressure and filling it in in the app. And, you know, it’s reassuring that if it is high or deviating, that someone (a health professional) is watching and will take action.”^31^	^12,16,21,31,34,38,41,42^
** Reducing the frequency of clinical appointments**	“I think it’s an important thing to have (the device) if we can afford because our visit to the doctor (is infrequent). Duration between visits is about 3 months.”^12^ “Even though it’s the doctors job because of course, and different things in the medical centers I would have thought maybe just saving time for more serious things and other people.”^40^ “I hate going to the doctors, and you can do it you know whenever you want at home … it’s so much easier.”^19^	^12,16,18–20,31,33,36,38–40^
**Triggering confusion and stress**		
** Anxiety and panic over “bad” numbers**	“I say if the person that doing it know he has a high reading then he will worry, but if you got somebody who don’t know they would worry them because they don’t know, because it’s high they don’t know, they don’t care and then they die.”^25^ “I felt it was intrusive. I started worrying about my BP.”^28^ “I think having it at home and monitoring it would be a lot less stressful than having to go out and have it monitored.”^39^	^14,18,19,28,36,38–40^
** Constant reminder of illness identity**	“I just don’t want to be too aware of doing it.”^43^ “(self-blood pressure monitoring) might put too much emphasis on (patients) condition and (patients) do not want to be confronted with it too much”.^22^	^24,43^
** Disregarded by clinicians**	“But when we went for checkup, the doctor rarely tells us the actual blood pressure reading. He did not tell us the readings he just said it’s okay. Actually we would like to know, the actual reading of the diastolic blood pressure.”^12^ “With blood pressure you’d put it in the chart then and you would go back to the GP but you need the feedback because I could wait and wait or it’s going somewhere into a pile and it’s a waste of time that you are doing it.”^25^ “I think intrinsically (doctors) were a bit sort of dubious about whether I could do it myself and whether I would be doing it accurately.”^19^	^12,15,19,23,25,26,29,36,38,43^
** Lack of confidence in interpreting and responding to results**	“I felt more confident that they’ve done it, rather than if I was doing it myself.”^19^ “Every time I measure, I wonder: do I measure technically good? Do I operate the product in the right way? Do I measure at the right moment?”^22^ “I don’t know what good it would do but I would ask about that you know, does my blood pressure readings over the next 12 months … what’s that going to do how is that going to help anybody because I can’t do nothing about my readings can I and it would concern me if it went up and down a little bit he would say well I am going to put you on stronger drugs.”^25^	^15,17,19,24,25,36,40,44^
** Redundancy of continuous monitoring**	“I just got bored with it was just a toy, that’s what I do, you know what I do I pop things and get bored... after 6 months.”^25^ “I thought that (self-monitoring blood pressure) would be very overwhelming to add on top of (activities to do) going home.”^39^	^15,18,24,25,39,43,46^
** Uncertainties around targets and frequency of measures**	“Because my previous readings (systolic blood pressure [SBP]) are 179, 180 (mm Hg) and the lower reading 110 (mm Hg). So, when I get reading of (SBP) 159 (mm Hg) and occasionally the lower reading (diastolic blood pressure) 90 (mm Hg), I felt happy. So, for me when I occasionally get the reading (SBP) 140 (mm Hg), that is already very good for me.”^12^ “The frequency of checking blood pressure also varied. Some measured their blood pressure daily without fail. Others did it only when they had symptoms or when it was convenient. None had written the readings down in a record book. I checked mine every day, without miss. One time before going to sleep.”^12^ “Where should it (BP) be? That way I’ll know if me or the missus gotta give a call to an ambulance to come get me...because I don’t know what it’s supposed to be. I don’t know if I should call.”^42^	^12,40,42–44^
** Concerns of unreliability**	“That’s why. I am also very suspicious, I mean, (the readings are) so variable within 2 weeks you know. Up and down, never consistent. So, we wonder whether I should be taking tablets. Is it worth of taking tablets or is it worth of using the machine at all, you see.”^12^ “Not comfortable to test at home (…) worried about making mistakes and to have inaccurate readings.”^36^	^12,22,32,34,36,43^
**Financial and operational burden of device**		
** Vulnerability preventing use**	“I don’t understand what this number means. I can’t figure it out because I don’t know which figure refers to high blood pressure. What is low blood pressure? I have no idea. I need more time to understand this because I am so forgetful. I sometimes forget things.”^34^ “We are old, we cannot remember how to operate the machine, even though the support staff showed us the procedure three times. We need to operate this equipment on our own! We need to be familiar with the machine, but unlike young people who can learn quickly and remember it forever, this is just not for the elderly.”^34^	^19,31,34^
** Unsustainable cost**	“I think the device needs to be checked to ensure that it is working properly; if it needs to be repaired frequently, it would be a barrier to use. It is not possible for me to pay for it.”^34^ “To check at home … you will find that others don’t have money to buy the monitor.”^36^	^15,19,34,36^

### Enabling autonomy and empowerment of own health

*Allowing access to comprehensive and accurate data*. Patients found that regular monitoring of blood pressure provided objective data about trends in their blood pressure readings. Self-monitoring of blood pressure allowed them to be *“*able to interpret (results) correctly.”^31^ They believed this information supported better communication with their clinicians about their blood pressure. Patients believed that self-monitoring of blood pressure “confirmed blood pressure readings which were measured at the clinic”^15^ and helped them receive “quick responses” regarding their blood pressure.^19^ Some mentioned that the readings taken by their clinicians could be inaccurate due to anxiety and discomfort leading to higher blood pressure readings*—*“I get a more accurate reading when I’m not stressed when I’m at home.”^19^

*Bolstering motivation for lifestyle changes.* Patients felt a stronger sense of responsibility while monitoring their blood pressure, and were motivated to improve their blood pressure levels through lifestyle changes and setting targets for blood pressure. They felt more proud, excited, and satisfied in having the autonomy to manage their condition and “more confident where (they) have had to speak to (doctors) about (blood pressure).”^19^

*Encouraging diligence in medication taking.* Patients were motivated to take antihypertensive medications diligently upon seeing lower blood pressure readings after taking medications—“You can see whether the medicine is effective.”^12^ Patients voiced that “seeing those high numbers”^14^ on home blood pressure monitoring devices prompted them to take medications to avoid serious consequences.

*Gaining interest in self-management.* Monitoring blood pressure at home allowed patients to take responsibility in managing their health—“It’s important for me to be more intentional and more in with my healthcare.”^32^ Patients also used their blood pressure readings to negotiate the management of hypertension with their general practitioners (GPs) and “take me off (medications).”^43^

*Increasing awareness of health status.* With home blood pressure monitoring, participants felt they gained awareness about their blood pressure and what numbers meant. Patients reported that checking their blood pressure frequently made them “aware of the high readings and encourage them to seek medical help.”^36^ Patients gave more attention to blood pressure and were prompted to think about their disease more regularly.

### Providing reassurance and convenience

*Instilling a sense of security.* Patients felt reassured in being able to monitor blood pressure at home and in knowing their blood pressure readings were stable—“I get peace of mind to know that my blood pressure is more or less (doctors) say on a level.”^25^ This was particularly relevant for those who waited long periods of time between clinical appointments—“Because of this device, I feel at ease.”^34^

*Readiness for troubleshooting.* Patients expressed relief in being able to check their blood pressure when they were feeling unwell or had symptoms. This allowed them to self-assess and take action to manage their blood pressure. They also thought that early flagging of symptoms related to hypertension was possible with blood pressure monitoring, and as such helped to get “psychological relaxation.”^12^

*Reducing the frequency of clinical appointments*. Patients reported a reduction in the frequency of routine follow-up or urgent visits. This was particularly relevant for those who lived in remote areas, had mobility issues, or lacked resources and time—“Having (blood pressure device) at home would be a lot less stressful than having to go out and have it monitored.”^39^ Others reported discomfort in sitting in the waiting room—“It was really uncomfortable to sit for half an hour, so by the time I got upstairs, my blood pressure was high.”^19^

### Triggering confusion and stress

*Anxiety and panic over “bad” numbers*. Some patients felt that home blood pressure monitoring was intrusive, triggering feelings of anxiety when numbers were high or there were fluctuations in readings—“If you get a bit of a neurotic person you could end up with (taking readings) up every 5 minutes.”^40^

*Constant reminder of illness identity*. Some did not want to measure blood pressure to avoid being constantly reminded of their condition—“I don’t want to be too aware of doing it.”^43^ They avoid any negative associations with the readings, and “they do not want to be confronted with (hypertension) too much.”^22^

*Disregarded by clinicians*. Some patients reported that GPs had not offered them the option to monitor blood pressure at home.^15^ People felt ignored when clinicians did not pay attention to their home blood pressure readings or did not trust them to self-monitor blood pressure—“Intrinsically (doctors) were a bit sort of dubious about whether I could do it myself accurately.”^19^ Some lost motivation to persist with self-monitoring and felt disempowered because they perceived self-monitoring as a “waste of time,”^25^ if their clinicians did not give “feedback”^25^ on their readings.

*Lack of confidence in interpreting and responding to results*. Some were uncertain about the target ranges and frequency of monitoring. Patients were hesitant about taking responsibility for interpreting and responding to blood pressure measurements because “they are unfamiliar with the machine.”^36^ Some chose not to take their medications when they saw fluctuations in their blood pressure readings because they felt uncertain about their benefits—“I have to take the same (medication). I checked my reading: not different from what it was. Then should I stop?.”^12^

*Redundancy of continuous monitoring.* Some believed that daily monitoring of blood pressure was redundant because blood pressure was already being managed by their GPs. Patients felt that continuous monitoring could make people “hypochondriac.”^19^ Some thought that self-monitoring of blood pressure added more responsibilities in their daily lives and “it would be overwhelming.”^39^ Some felt “bored”^25^ with continuous monitoring. Some remarked that if patients “are adherent to medications, there was no need to check blood pressure.”^15^

*Uncertainties around targets and frequency of measures.* Some patients did not know the recommended target blood pressure. Others believed that the target blood pressure was individualized, and the goal was to achieve lower blood pressure compared to the previous readings—“Because my previous readings are 180 (mm Hg), when I get reading of 159 (mm Hg), I felt happy.”^12^ Patients were uncertain about the frequency of measuring their blood pressure—“Some measured their blood pressure daily without fail, others did it only when they had symptoms.”^12^

*Concerns of unreliability*. Some were concerned about the reliability of their home blood pressure monitoring readings—“I worried about making mistakes and to have inaccurate readings.”^36^ This was particularly the case when their home blood pressure measurements varied substantially from clinic measurements, or when there was considerable variability between readings. Some were concerned about mechanical problems and had difficulties in trusting a machine.

### Financial and operational burden of device

*Vulnerability preventing use.* For some patients, particularly those with advanced disease, physical limitations, impaired eyesight, or the elderly, the use of home blood pressure monitoring devices was too challenging and burdensome because they felt too weak to use the device, or were highly dependent on others for self-management tasks. Also, those with limited technology and health literacy found it difficult to use—“I don’t understand what this number means.”^34^

*Unsustainable cost.* Patients in low resources settings were not able to afford blood pressure monitoring devices, and including those who installed home telemonitoring systems where there would be ongoing costs involved for software—“Patients with this disease usually have already purchased their own sphygmomanometer, and this device probably is not cheap either!.”^34^ However, patients also voiced that a shift to self-monitoring of blood pressure could reduce burden and cost on the healthcare system.

## Discussion

For patients with hypertension, self-monitoring of blood pressure encouraged autonomy and empowerment of their own health. Self-monitoring of blood pressure provided patients with a sense of safety and helped them to be prepared for managing their symptoms. On the other hand, some patients reported that self-monitoring of blood pressure triggered stress and confusion due to uncertainty in interpreting results. They were also concerned that the blood pressure monitoring device was unaffordable or too complex.

While many of the themes were similar across the studies conducted in different populations and settings, there were apparent differences in perspectives based on age and resource settings. Older patients raised concerns around operating the device and were unfamiliar with and reluctant to engage with technological devices. Some participants in low-resource settings noted that they could not afford the devices due to the financial burden or purchasing the equipment.

Previous studies have found that patients with hypertension have expressed that self-monitoring of blood pressure provided more accurate readings compared with the clinical assessment of blood pressure, and was easy to use.^47^ People with hypertension who self-monitor blood pressure felt more inclined to adopt lifestyle changes^48^ and were confident in communicating their readings to clinicians.^49^ However, other patients with hypertension felt confused about the utility of home blood pressure monitoring, particularly if they were not aware of how to interpret the results.^47^ Previous work has also highlighted challenges including inaccuracy in self-monitoring blood pressure,^50^ patients being unaware about targets,^49^ or lack of training from clinicians.^51^ Our synthesis suggests that patients were more engaged and had a sense of achievement in taking control of their care, and felt reassured in being able to monitor blood pressure at home between clinical appointments. On the other hand, some patients felt that continuous monitoring of blood pressure was a constant reminder their condition, and could potentially lose motivation if they felt that clinicians did not provide feedback or expressed doubt about the patients’ ability to self-monitor blood pressure.

In our systematic review, we conducted a comprehensive search, assessed the transparency of study reporting, and used an explicit framework to assess and synthesize the findings. We used investigator triangulation to ensure that we captured the breadth and depth data across the included studies. However, our study has some potential limitations. We only included studies published in English, and most studies were conducted in high-income countries, which may limit the generalizability of our findings We have also included people with either gestational hypertension/preeclampsia or on dialysis (including both hemodialysis and peritoneal dialysis) and these populations may differ from other hypertensive patients, thus the transferability the findings from these studies may need to be considered in the context of the patient population. The home blood pressure monitoring was evaluated using different devices across the studies included, and this may prevent our capability to evaluate possible differences among patient experiences and expectation.

This study identified potential areas of relevance to clinical practice. Patients identified that home-based self-monitoring of blood pressure was valuable in identifying white coat or masked hypertension, and helped them in adopting protective behaviors such as higher adherence to drugs, lifestyle changes, and early warning to inform clinicians regarding fluctuations in readings to prevent complications. However, patients identified limitations in their understanding, training, and a need for technical support. We suggest that clinicians provide education about blood pressure targets, frequency, how to read and interpret the results.^52^ There may also be a need to reassure the patients and manage the psychological impacts, including anxiety of receiving readings that are outside the recommended range, and discussing impacts on illness identity and depression.^53^ Addressing uncertainties about correct procedures, inaccuracy of the readings, and technical issues in operating home blood pressure monitoring may also reduce patient concerns.^54^

Incorporating self-monitoring of blood pressure into digital health technologies has the potential to enhance the delivery of health care for the individual. Since the COVID-19 pandemic, there has been a surge in the use of telemedicine, where the results may be automatically sent to clinicians for review and inform treatment strategies for managing hypertension.^55^ There is also potential for e-health tools (e.g., apps) to promote behavioral change.^56,57^ However, as technology advances, it is important to also ensure that devices are optimized so vulnerable populations are not excluded from their use. For dependent patients, or those with visual impairment, our findings highlighted the need to improve the display of the results.^58^ We also advocate for financial assistance for patients in low-resource settings to access blood pressure devices.

Home blood pressure monitoring for hypertension management has been identified as a high-priority research question by patients and health professionals.^59^ Further research on interventions to improve self-monitoring of blood pressure should integrate patient perspectives and include outcomes of importance to patients. Based on the INVOLVE^60^ and Patient-Centered Outcomes Research Institute (PCORI)^61^ initiatives, which provide frameworks and recommendations for involving patients across all stages of research, we recommend that patients should be involved both in developing and evaluating interventions to strengthen uptake. Trials of blood pressure monitoring often assess surrogate outcomes, whilst mortality, cardiovascular events, and patient-reported outcomes (e.g., health-related quality of life)^62^ have been inconsistently reported. This limits the ability to evaluate the impact of self-monitoring of blood pressure on longer-term outcomes.^52,56^ Economic modeling studies have shown that home blood pressure monitoring is cost-effective for diagnosing and treating hypertension.^63^

Home blood pressure monitoring can increase patient motivation, empowerment, autonomy, and provide reassurance in managing hypertension. However, the challenges of uncertainty in readings, technical complexities, and cost remain. Education and support for patients may help to reduce the practical and psychological burden of blood pressure monitoring and may thereby improve patient outcomes.

## Supplementary Material

Supplementary materials are available at *American Journal of Hypertension* (http://ajh.oxfordjournals.org).

hpad021_suppl_Supplemental_Material

## Data Availability

No additional data are available.
