# Supplementary material for: Perspectives and Experiences of Self-monitoring of Blood Pressure Among Patients With Hypertension: A Systematic Review of Qualitative Studies
Source: Am J Hypertens. 2023 Feb 25;36(7):372–84. doi: 10.1093/ajh/hpad021 (PMC10267641; doi:10.1093/ajh/hpad021)
Supplement: hpad021_suppl_Supplemental_Material [file hpad021_suppl_supplemental_material.pdf]

Supplementary Files

Table S1: Search strategies

MEDLINE 1946 to March 08, 2022

1. qualitative$.tw.
2. exp qualitative research/
3. interview$.tw.
4. focus group$.tw.
5. (thematic$ or theme$).tw.
6. ethnograph$.tw.
7. phenomenon$.tw.
8. symbolic interact$.tw.
9. grounded theory.tw.
10. quality of life/
11. psychology, social/
12. anxiety/ or depression/
13. emotions/
14. satisfaction/
15. process evaluation$.tw.
16. exp Health Behavior/ or exp Health/
17. Exp Cardiovascular Diseases/
18. Exp Hypertension/
19. blood pressure$.tw
20. exp patients/
21. patient$.tw.
22. exp biosensing techniques/
23. (sensor or sensors or sensing$).tw.
24. (biosensor$ or biosensing$).tw.
25. exp Wearable Electronic Devices/
26. wearable$.tw.
27. or/1-16
28. or/17-21
29. or/22-26
30. 27 and 28 and 29

Embase 1974 to March 08, 2022

1. exp biosensor/
2. (sensor or sensors or sensing$).tw.
3. (biosensor$ or biosensing$).tw.

4. exp sensor/
5. wearable$.tw.
6. qualitative$.tw.
7. exp qualitative research/
8. interview$.tw.
9. focus group$.tw.
10. (thematic$ or theme$).tw.
11. ethnograph$.tw.
12. phenomenon$.tw.
13. symbolic interact$.tw.
14. grounded theory.tw.
15. quality of life/
16. exp social psychology/
17. emotions/
18. exp satisfaction/
19. Exp cardiovascular diseases/
20. Exp hypertension
21. blood pressure$.tw
22. exp patients/
23. patient$.tw.
24. or/1-5
25. Or/6-18
26. Or/19-23
27. 24 and 25 and 26

PsycINFO 1806 to March 08, 2022

1. (sensor or sensors or sensing$).tw.
2. (biosensor$ or biosensing$).tw.
3. wearable$.tw.
4. blood pressure$.tw
5. qualitative$.tw.
6. exp Qualitative Methods/
7. interview$.tw.
8. focus group$.tw.
9. (thematic$ or theme$).tw.
10. ethnograph$.tw.
11. phenomenon$.tw.
12. symbolic interact$.tw.
13. grounded theory.tw.
14. quality of life/
15. exp Social Psychology/
16. exp Emotions/

17. exp Life Experiences/
18. exp Patients/ or exp Medical Patients/
19. patient$.tw.
20. exp "Chronicity (Disorders)"/ or exp Health Behavior/ or exp Chronic Illness/ or exp Self-Management/ or exp Disease Management/
21. chronic$.tw.
22. cardiovascular disease$.tw.
23. Exp hypertension/
24. Or/1-4
25. Or/5-17
26. Or/18-23
27. 24 and 25 and 26

CINAHL March 08, 2022

(MH "Blood Pressure Devices+") OR (MH "Monitoring, Direct Pressure+") S3. (MH ”Hypertension+”)

(MH “Qualitative Studies+”)

Table S2. COREQ appraisal

Item	Abdulla 2011	Albrecht 2020	Allen 2019	Al-Rousan 2020	Aquino 2022	Bengtsson 2014	Bostock 2009	Cairns 2020	Carter 2021	Evangelidis 2021	Geerse 2019
Personal Characteristics											
Interviewer/facilitator identified	1	0	1	1	1	0	0	1	1	0	0
Occupation of the interview/facilitator	0	1	1	1	1	0	0	1	1	0	0
Experience or training in qualitative research	0	0	0	1	1	0	0	1	0	0	0
Relationship with participants											
Relationship established before study start	1	0	0	1	1	0	0	1	0	0	0
Participant selection											
Selection strategy	1	0	1	1	1	1	1	0	1	0	1
Method of approach or recruitment	1	0	1	0	1	1	1	1	1	0	0
Sample size	1	1	1	1	1	1	1	1	1	0	1
No. and/or reasons for nonparticipation	0	0	1	1	0	0	0	1	1	0	0
Setting											
Venue of data collection	1	1	1	1	1	1	1	1	1	0	0
Presence of nonparticipants (e.g. clinical staff)	0	0	1	0	0	0	0	1	0	0	0
Description of sample	1	1	1	1	1	1	1	1	1	1	1
Data collection											
Questions, prompts or topic guide	1	1	1	1	1	1	1	1	0	0	1
Repeat interviews/observations	0	0	1	0	1	0	0	0	0	0	0
Audio/visual recording	1	1	1	1	1	1	1	1	1	0	1
Field notes	1	0	0	0	0	0	1	1	0	0	1
Duration of data collection	0	1	0	1	1	0	0	1	1	0	0
Translation and interpretation	N/A	0	0	1	N/A	0	0	N/A	N/A	0	0
Data preparation and transcription	1	1	1	1	0	1	1	1	0	0	1
Data (or theoretical) saturation	1	0	1	1	1	1	0	1	1	0	0
Data Analysis											
Researcher/expert triangulation	1	0	1	1	1	1	1	0	1	1	1
Translation	N/A	0	0	1	N/A	0	0	N/A	N/A	0	0
Derivation of themes or findings	1	1	1	1	1	1	1	1	1	1	1
Use of software	1	1	1	0	1	1	0	1	1	1	0
Member checking	0	0	0	0	1	0	0	1	1	0	0
Reporting											
Participant quotations or raw data provided	1	1	1	1	1	1	1	1	1	1	1
Range of depth of insight into participant perspectives	1	1	1	1	0	1	0	1	0	0	1

Item	Glynn 2015	Grace 2017	Grant 2015	Halifax 2007	Hall 2014	Hanley 2013	Helou 2021	Jones 2012	Jongsma 2021	Koopman 2020	Lambert-Kerzener 2010	Lu 2013
Personal Characteristics												
Interviewer/facilitator identified	0	0	0	0	0	1	1	0	0	1	1	1
Occupation of the interview/facilitator	1	1	0	1	0	1	1	0	0	1	0	0
Experience or training in qualitative research	1	0	0	0	0	1	1	0	0	1	0	0
Relationship with participants												
Relationship established before study start	1	0	0	0	0	0	1	0	0	0	0	0
Participant selection												
Selection strategy	1	0	1	1	1	1	1	1	1	1	1	1
Method of approach or recruitment	0	0	1	1	0	1	1	0	1	1	0	1
Sample size	1	1	1	1	1	1	1	1	1	0	1	1
No. and/or reasons for nonparticipation	0	1	0	0	1	0	1	1	0	0	0	0
Setting												
Venue of data collection	1	1	1	0	0	1	1	1	1	0	1	0
Presence of nonparticipants (e.g. clinical staff)	1	0	0	0	0	0	1	0	0	0	0	0
Description of sample	1	1	1	1	1	1	1	1	1	1	1	1
Data collection												
Questions, prompts or topic guide	1	1	1	0	1	1	1	1	1	1	1	1
Repeat interviews/observations	0	0	0	0	0	0	1	0	0	0	0	0
Audio/visual recording	1	1	1	1	1	1	1	1	1	0	1	1
Field notes	0	0	1	0	0	0	1	1	0	1	0	0
Duration of data collection	0	0	0	1	0	1	1	1	0	0	0	1
Translation and interpretation	0	N/A	0	N/A	0	0	N/A	0	0	0	0	0
Data preparation and transcription	1	1	1	1	1	1	1	1	1	1	1	1
Data (or theoretical) saturation	1	0	1	0	0	1	1	1	1	0	0	0
Data Analysis												
Researcher/expert triangulation	1	1	1	1	1	1	1	1	1	1	1	1
Translation	0	N/A	0	N/A	0	0	N/A	0	0	0	0	0
Derivation of themes or findings	1	1	1	1	1	1	1	1	1	1	1	1
Use of software	1	1	1	0	1	1	1	0	1	1	1	0
Member checking	1	0	1	0	0	1	0	0	1	1	0	0
Reporting												
Participant quotations or raw data provided	1	1	1	0	1	1	1	1	1	1	1	1
Range of depth of insight into participant perspectives	1	0	1	0	0	1	0	1	1	1	0	0

Item	McBride 2020	Munyungula 2021	Ondienge 2017	Ovaisi 2011	Payakachat 2020	Rickerby 2003	Rohela 2021	Schmid 2009	Tompson 2018	Vasileiou 2013	Ware 2018	Xiao 2019
Personal Characteristics												
Interviewer/facilitator identified	1	0	0	0	0	0	0	0	0	1	1	0
Occupation of the interview/facilitator	1	0	0	1	0	1	0	0	1	0	0	1
Experience or training in qualitative research	1	0	0	0	0	0	0	0	1	0	0	1
Relationship with participants												
Relationship established before study start	1	0	0	0	1	0	1	0	1	0	0	0
Participant selection												
Selection strategy	1	1	1	1	0	1	1	1	1	1	1	1
Method of approach or recruitment	1	1	0	0	0	1	1	1	1	1	0	0
Sample size	1	1	0	1	1	1	1	1	1	1	1	1
No. and/or reasons for nonparticipation	1	0	0	1	0	1	1	0	1	1	0	0
Setting												
Venue of data collection	1	0	0	0	1	1	1	0	1	1	1	1
Presence of nonparticipants (e.g. clinical staff)	1	0	0	0	0	0	0	0	1	0	0	1
Description of sample	1	1	0	1	1	1	1	1	1	0	1	1
Data collection												
Questions, prompts or topic guide	1	1	0	1	0	1	0	1	1	1	1	1
Repeat interviews/observations	1	0	0	0	0	0	0	0	0	0	0	0
Audio/visual recording	1	1	1	1	1	1	1	1	1	1	1	1
Field notes	1	1	0	1	0	1	0	0	0	0	0	0
Duration of data collection	1	1	0	1	1	0	0	0	1	0	0	1
Translation and interpretation	0	0	N/A	0	N/A	0	N/A	0	0	N/A	0	0
Data preparation and transcription	1	0	1	1	0	1	0	1	1	0	1	0
Data (or theoretical) saturation	1	1	0	0	0	1	0	0	1	1	1	0
Data Analysis												
Researcher/expert triangulation	1	0	0	1	1	1	1	1	1	1	1	1
Translation	0	0	N/A	0	N/A	0	N/A	0	0	N/A	0	0
Derivation of themes or findings	1	0	1	1	1	1	1	1	1	1	1	1
Use of software	1	0	1	1	1	0	1	1	1	1	1	1
Member checking	1	0	0	0	0	0	0	0	1	0	0	0
Reporting												
Participant quotations or raw data provided	1	1	1	1	1	1	1	1	1	1	1	1
Range of depth of insight into participant perspectives	0	1	1	0	1	0	0	1	1	0	0	0
